# Increased Iron Sequestration in Alveolar Macrophages in Chronic Obtructive Pulmonary Disease

**DOI:** 10.1371/journal.pone.0096285

**Published:** 2014-05-01

**Authors:** Quentin Philippot, Gaëtan Deslée, Tracy L. Adair-Kirk, Jason C. Woods, Derek Byers, Susan Conradi, Sandra Dury, Jeanne Marie Perotin, François Lebargy, Christelle Cassan, Richard Le Naour, Michael J. Holtzman, Richard A. Pierce

**Affiliations:** 1 Division of Pulmonary and Critical Care Medicine, Department of Internal Medicine Washington University, St. Louis, Missouri, United States of America; 2 Institut National de la Santé Et de la Recherche Medicale Unit 903, University Hospital, Reims, France; 3 Department of Pulmonary Medicine, University Hospital of Reims, France; 4 Department of Radiology, Washington University, St. Louis, Missouri, United States of America; 5 EA4683, University of Reims, Reims, France; University of Giessen Lung Center, Germany

## Abstract

Free iron in lung can cause the generation of reactive oxygen species, an important factor in chronic obstructive pulmonary disease (COPD) pathogenesis. Iron accumulation has been implicated in oxidative stress in other diseases, such as Alzheimer’s and Parkinson’s diseases, but little is known about iron accumulation in COPD. We sought to determine if iron content and the expression of iron transport and/or storage genes in lung differ between controls and COPD subjects, and whether changes in these correlate with airway obstruction. Explanted lung tissue was obtained from transplant donors, GOLD 2–3 COPD subjects, and GOLD 4 lung transplant recipients, and bronchoalveolar lavage (BAL) cells were obtained from non-smokers, healthy smokers, and GOLD 1–3 COPD subjects. Iron-positive cells were quantified histologically, and the expression of iron uptake (transferrin and transferrin receptor), storage (ferritin) and export (ferroportin) genes was examined by real-time RT-PCR assay. Percentage of iron-positive cells and expression levels of iron metabolism genes were examined for correlations with airflow limitation indices (forced expiratory volume in the first second (FEV_1_) and the ratio between FEV_1_ and forced vital capacity (FEV_1_/FVC)). The alveolar macrophage was identified as the predominant iron-positive cell type in lung tissues. Futhermore, the quantity of iron deposit and the percentage of iron positive macrophages were increased with COPD and emphysema severity. The mRNA expression of iron uptake and storage genes transferrin and ferritin were significantly increased in GOLD 4 COPD lungs compared to donors (6.9 and 3.22 fold increase, respectively). In BAL cells, the mRNA expression of transferrin, transferrin receptor and ferritin correlated with airway obstruction. These results support activation of an iron sequestration mechanism by alveolar macrophages in COPD, which we postulate is a protective mechanism against iron induced oxidative stress.

## Introduction

Iron is critical for the maintenance of cell homeostasis, having important roles in respiration, DNA synthesis, energy production, and metabolism. However, excess iron can be detrimental because of its potential to generate harmful free radicals. Because of this, tight regulation of iron metabolism is essential. Perturbation from normal physiologic iron concentrations has been associated with the pathogenesis of aging, neurodegenerative disease,and cancer [1], [2], presumably via the generation of excess reactive oxygen species (ROS). The role of iron in other diseases in which oxidative stress has been implicated remains to be determined.

Chronic obstructive pulmonary disease (COPD), comprised of irreversible airways obstruction and alveolar space enlargement or emphysema, is a major cause of mortality and morbidity worldwide [3]. Cigarette smoke is the main etiological factor of COPD [3], which triggers an inflammatory response in the lung. Oxidative stress induced by the free radicals in tobacco smoke and produced by inflammatory cells has been strongly implicated in the pathogenesis of COPD. In addition, excess iron accumulation in the lung has been reported in association with cigarette smoke [4]–[6] and severe emphysema [7]. Moreover, cigarette smoke can alter lung iron metabolism in animal models [8]. However, it is unknown where iron accumulates in lungs of COPD subjects, if expression of iron uptake and storage genes in the lung differs between controls and subjects with COPD, and whether changes in iron metabolism correlate with disease severity.

This study sought to 1) quantify the iron deposits in the lung tissue of lung transplant donors, GOLD 2–3 (moderate to severe COPD), and GOLD 4 (very severe COPD) subjects, and in bronchoalveolar lavage (BAL) cells from smokers, non-smokers, and GOLD 1–3 COPD subjects, 2) identify the iron-accumulating cell types in the lung parenchyma, 3) determine the expression of transferrin and transferrin receptor (iron uptake), ferritin (iron storage) and ferroportin (iron export), and 4) determine correlations of changes in iron metabolism gene expression with airflow limitation indices (forced expiratory volume in the first second (FEV_1_) and the ratio between FEV_1_ and forced vital capacity (FEV_1_/FVC)) which are indicative of COPD severity.

## Materials and Methods

### Ethics Statement

The lung parenchyma study was approved by the Human Studies Committee of Washington University and the bronchoalveolar lavage study was approved by the Institutional Review Board of the University Hospital of Reims.

### Subjects, Lung Processing, Sampling, and Collection of BAL

Lung samples were obtained from 20 GOLD 4 COPD subjects receiving lung transplant, 9 GOLD 2–3 COPD subjects undergoing resection of lung cancer (avoiding areas affected by tumor), and 8 non-COPD lung donors obtained following size adjustment for transplantation as controls. The lungs were processed as previously described [9]. BAL samples were obtained from a second set of non-cancer, GOLD 1–3 COPD subjects, healthy smokers and healthy non smokers who underwent fiberoptic bronchoscopy according to American Thoracic Society recommendations [10]–[12]. Briefly, BAL was performed by instilling saline solution in a sub-segmental bronchus, followed by aspiration and discarding of the first 50 ml aliquot. The remaining BAL fluid was centrifuged and cells were used for this study. In both subject sets, COPD diagnosis and GOLD classification was based on spirometric pulmonary function tests according to the Global Initiative for Chronic Obstructive Lung Disease consensus statement[3], and informed, written consent was obtained from each subject.

### Histochemical Staining

Ferric iron was detected on serial 5-µm paraformaldehyde-fixed, paraffin-embedded lung sections using Perls DAB staining. Briefly, slides were incubated for 30 min in 5% potassium ferrocyanide (Sigma-Aldrich, St. Louis, MO) and 5% hydrochloric acid, followed by a 15 min incubation in DAB (Peroxidase Substrate Kit; Vector Laboratories, Burlingame, CA). Negative control slides were incubated for 30 min in PBS and 15 min in DAB. Immunohistochemical staining for macrophages, transferrin or ferritin was performed using a Vectastain kit (Vector Laboratories; Burlingame, CA) using mouse anti-CD68 (1∶100, KP1; Dako, Kyoto, Japan), rabbit anti-transferrin (1∶30 000, A-0061; DakoCytomation, Carpinteria, CA), or anti-H-ferritin (1∶2000, F-5012; Sigma) antibodies, respectively. Isotype-matched, nonimmune immunoglobulins served as negative controls.

### Quantification and Identification of Iron-positive Cells

Lung sections were stained with Perls-DAB for iron content (brown-black color) and anti-CD68 (red color) to identify macrophages. Dark staining anthracotic material and macrophages with dark-colored content were assessed on a consecutive serial section stained with nuclear fast red only (American Master Tech Scientific Inc., St. Lodi, CA). Slides were scanned using a NanoZoomer 2.0 (Hamamatsu Photonics, K.K., Japan) and the staining was quantified using Image-Pro Plus Software (MediaCybernetics, Silver Spring, MD). The area of cells positive for iron was calculated using the following formula: *Iron positive cell area  =  (Area of brown-black color on Perls-DABstained slide – Area of brown-black color on the nuclear fast red slide)/(Area of pink color on the nuclear fast red slide) x 100*. The percentage of iron-positive macrophages was determined on Perls-DAB-CD68 co-stained slides from 3 randomly selected 10x fields containing ≥10 macrophages per field.

### RNA Isolation and Quantitative Real-time RT-PCR

Total RNA was isolated from human donor and GOLD 4 COPD lung tissue samples, and BAL cells using TRIzol reagent (Invitrogen, Carlsbad, CA). Quantitative Real-Time RT-PCR was performed as follow: the cDNA was synthesized using SuperScript II reverse transcriptase (Invitrogen). Real-time RT-PCR employed the Fast SYBR Green Master Mix (Applied Biosystems, Foster City, CA) and gene-specific primers (Table S1) on an Eco™ Real-Time PCR System (Illumina, San Diego, CA).Results were standardized using the delta-delta C_T_ method [13] using the average of the expression GAPDH, HRPT1 (Hypoxanthine phosphoribosyltransferase-1) and PPIA (Peptidylprolyl isomerase-1) for normalization [14], [15].

### Morphological Analysis

For 14 GOLD 4 COPD and 4 non-COPD subjects, CT-scan of the frozen lungs was performed, and analyzed as previously described [9]. Briefly, the mean radiograph attenuation, expressed in Hounsfield Units (HU), was determined in the CT section corresponding to the lung area of the tissue samples using a separate image processing program (ImageJ; available at: http://rsb.info.nih. gov/ij).

### Statistical Analysis

A Student’s t test was used to compare between two groups, an Anova analysis with a Tukey-Kramer post-hoc test was used to compare between more than two groups and Spearman rank correlation was used to test for correlations between variables with a p≤0.05 considered significant.Statistical analysis was performed using Excel 2011 (Microsoft Corporation, Redmond, WA).

## Results

### Patient Characteristics

Peripheral lung tissue was obtained from 20 GOLD 4 COPD subjects receiving lung transplants for severe emphysema (GOLD 4), 9 GOLD 2***–***3 COPD subjects undergoing resection of lung cancer (avoiding areas affected by tumor), and 8 non-COPD donor lungs as controls. Their clinical and demographic characteristics are displayed in Table 1. As expected, the donor group consisted of younger, non-smokerssubjects. Pulmonary function tests were not obtained from donors.

**Table 1:** Clinical characteristics of subjects included in lung parenchyma study.

Characteristic	Donor	GOLD 2–3 COPD	GOLD 4 COPD
No. of subjects	8	9	20
Age	42±18	62±11*	59±6*
Sex, M/F	4/3 (7/8)	4/5	7/14
Smoking Pack-years	0±0	41±31	55±27
FEV1 (% of predicted)	-	62±17	18±4#
FEV1:FVC	-	57±15	31±9#
BMI (kg/m^2^)	-	28±5	23±3#

*p<0.05 between donor and spirometric GOLD 2–3 or GOLD 4 COPD,

# p<0.05 between spirometric GOLD 2–3 and GOLD 4 COPD.

In addition to lung tissue samples, BAL cells were obtained from another set of subjects: 8 healthy non-smokers, 8 healthy smokers, and 10 GOLD 1***–***3 COPD subjects. Their clinical and demographic information are presented in Table 2. As expected, subjects with COPD had increased airflow limitation compared to the healthy non-smokers and the healthy smokers groups. The BAL differentials are also presented in Table 2. The total number of inflammatory cells in the BAL fluid of smokers and COPD subjects were higher than non-smokers. However, in each group the predominant cell type (almost 90%) in the BAL were macrophages.

**Table 2:** Clinical characteristics of subjects included in BAL study.

Characteristic	Non Smokers	Smokers	GOLD 1–3 COPD
No. of subjects	8	8	10
Age	54±15	45±11	57±7
Sex, M/F	5/3	4/4	8/2
Smoking Pack-years	0±0	39±16*	45±13*
FEV_1_ (% of predicted)	96±13	87±5	65±16#
FEV_1_/FVC (%)	81±6	78±9	62±9#
BMI (kg/m^2^)	27±3	27±10	26±5
**Treatments (% of patient using the treatment):**			
Inhaled LABA	1/8	0/8	5/10
Inhaled SABA	0/8	0/8	1/10
Inhaled LAMA	0/8	0/8	3/10
Inhaled CS	1/8	0/8	4/10
Oral CS	0/8	0/8	1/10
**Cellular content of BAL**			
Macrophages (%)	87±6	86±9	90±5
Lymphocytes (%)	10±6	7±6	6±3
Neutrophils (%)	2±2	7±8	4±4
Eosinophils (%)	1±2	0±0	0±1
Total cell number per ml	177 571	412 875	237 000

*p<0.05 between smokers or COPD and non smokers,

# p<0.05 between non smokers or smokers and COPD, LABA = Long-Acting β-Agonists, SABA: Short-Acting β-Agonists., LAMA: Loan-acting muscarinic antagonists, CS: corticosteroids.

### Increased Iron Deposition in Severe COPD Lungs

**Figure 1:**
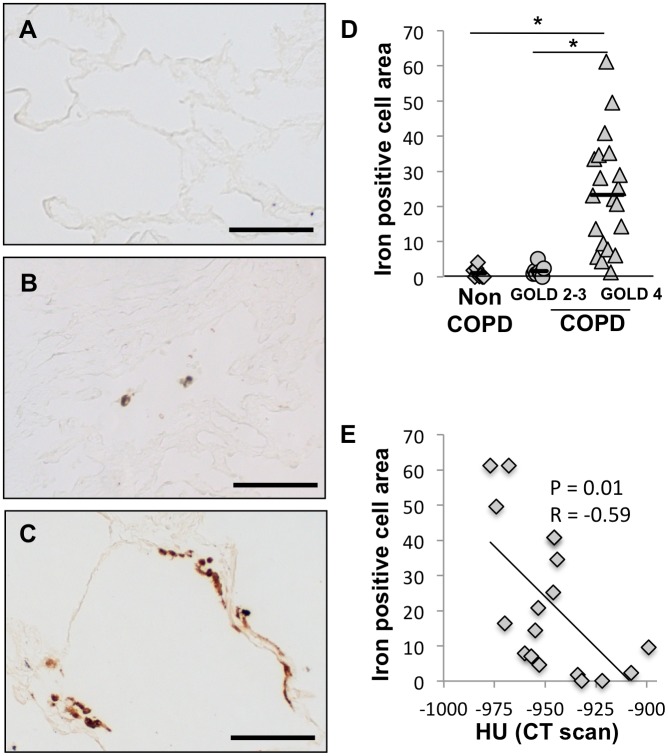
Iron deposits are increased in lungs of COPD subjects. Iron deposits were stained with Perls-DAB staining in lung samples obtained from (A) a patient without COPD, (B) a patient with GOLD 2 COPD and (C) a patient with GOLD 4 COPD. (D) Quantification of iron positive cellular area was performed on lung sections from 8 subjects without COPD, 9 subjects with GOLD 2 or 3 COPD and 20 subjects with GOLD 4 COPD. (E) Correlation between the iron deposits and the mean radiograph attenuation in the same lung area. *p<0.05 (Anova analysis with a Tukey-Kramer post-hoc test); Scale bars = 125 µm.

Iron accumulation in the lung was examined in 20 GOLD 4 COPD, 9 GOLD 2–3 COPD, and 8 non-COPD lungs by Perls-DAB staining. To distinguish between iron deposits and other dark anthracotic material, consecutive serial sections were stained with nuclear fast red only. To quantify the iron deposits, we calculated the iron-positive cell area by taking the area of brown-black color on Perls-DAB stained slide minus the area of brown-black color on the nuclear fast red stained slide divided by the area of pink color on the nuclear fast red slide. Iron deposits were rarely found in non-COPD lung parenchyma (Fig. 1A) but were detectable in the parenchyma of GOLD 2–3 COPD lungs and appeared abundant in GOLD 4 COPD lungs (Fig. 1B and 1C, respectively). The iron positive cell area was significantly increased in severe GOLD 4 COPD lungs (23±16%) compared to non-COPD (1.1±1.5%, p = 1.9×10^−5^) or GOLD 2–3 COPD (1.6±1.5%, p = 1.9×10^−5^) lungs (Fig. 1D). Moreover, iron positive cell area was found to correlate with the mean radiograph attenuation at the level of the lung tissue sample which is an index of emphysema severity (Fig. 1E). Our data suggest that excess iron accumulation is also associated with COPD and emphysema severity, with an increase in lung iron content in GOLD 4 COPD relative to GOLD 2–3 COPD subjects despite similar smoking pack years.

### Iron is Localized in Macrophages in COPD Lungs

**Figure 2:**
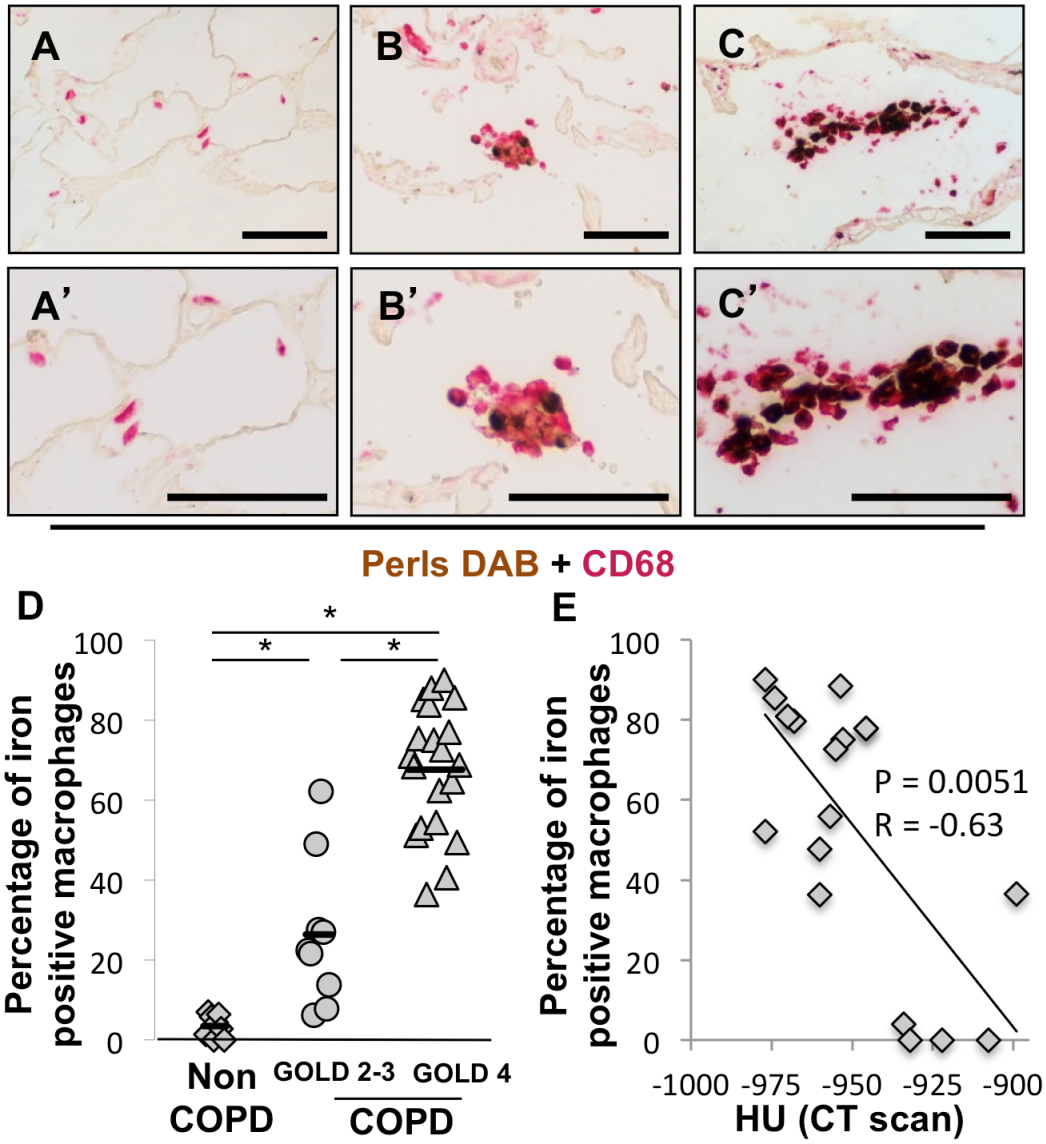
Iron is localized in macrophages in lung of COPD subjects and the percentage of iron positive macrophage increases with severity of disease. Perls-DAB and CD68 costaining was performed in lung samples obtained from (A–A’) a patient without COPD, (B–B’) a patient with grade 2 COPD and (C–C’) a patient with grade 4 COPD. (D) Percentage of iron positive macrophages was assessed in 8 lungs from subjects without COPD, 9 lungs from subjects with GOLD 2 or 3 COPD and lungs from 20 subjects with GOLD 4 COPD. (E) Correlation between the percentage of iron positive macrophages and the mean radiograph attenuation in the same lung area. *p<0.05 (Anova analysis with a Tukey-Kramer post-hoc test); Scale bars = 125 µm.

To quantify the prevalence of iron-positive macrophages, we performed co-staining of Perls-DAB and CD68 in lung sections. As shown in Figure 2B/B’ and 2C/C’, iron co-localized with macrophages in GOLD 2–3 and GOLD 4 COPD lungs, respectively, but not in non-COPD lungs (Fig. 2A/A’). The percentage of iron-positive macrophages was increased in GOLD 2–3 COPD lungs (26% ±19, p = 6.1×10^−12^) and GOLD 4 COPD lungs (68±16%, p = 6.1×10^−12^) compared to non-COPD lungs (3.5±2.8%). Interestingly, the persentage of iron-positive macrophages correlated with the mean radiograph attenuation of the lung tissue sample (Fig. 2E). These data suggest that the percentage of iron-positive macrophage increases with the severity of COPD and emphysema.

### Increased Expression of Iron Uptake Genes in COPD Lungs

Iron metabolism needs to be tightly regulated due to potential harmful effects of excess free iron. Free iron is bound by transferrin, taken into cells by the transferrin receptor, and is stored in cells bound to ferritin. Iron can be exported from cells via ferroportin. The expression of these genes is tightly regulated via the iron-responsive proteins (IREBs), which are able to interact with 5′ or 3′ untranslated region of their mRNA [16]. Among the two orthologous, IREB 2 has been associated with airflow limitation in GWA studies [17]–[19].

**Figure 3:**
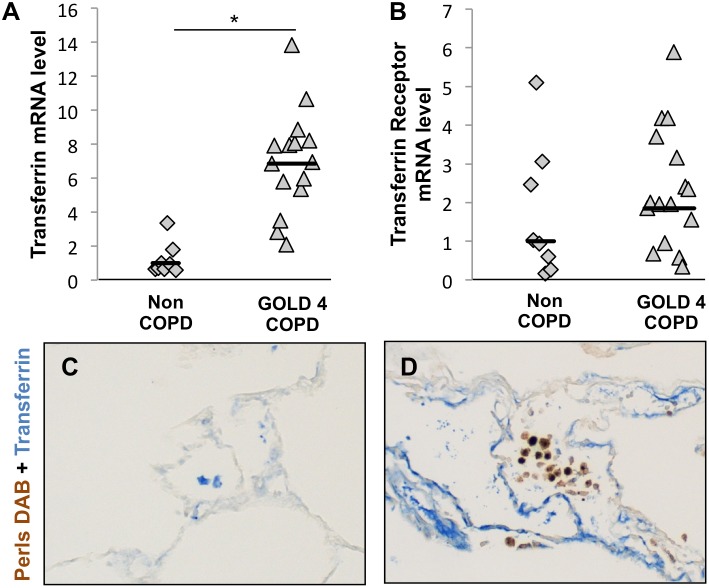
Iron uptake capacities are altered in lungs of COPD subjects. mRNA expression of (A) transferrin and (B) transferrin receptor was investigated in whole-lung total RNA samples obtained from 8 subjects without COPD and 16 subjects with GOLD 4 COPD. Three reference genes (GAPDH, HPRT1 and PPIA) were used for normalization. Costaining for transferrin and iron deposits, by Perls-DAB staining, was performed on lung samples obtained from (C) a subject without COPD and (D) a subject with GOLD 4 COPD. *p<0.05 (Student’s t test), Scale bars = 125 µm.

To determine wether the increased iron accumulation in COPD alveolar macrophages was a result of an increase in the expression of mRNAs encoding iron uptake proteins, we assessed the expression of transferrin and the transferrin receptor by real-time RT-PCR using RNA from 8 non-COPD lungsamples and 16 GOLD 4 COPD lungsamples. Transferrin expression was significantly increased in GOLD 4 COPD lungs compared to non-COPD lungs (fold increase = 6.9, p = 5.4×10^−6^, Fig. 3A). There was no significant difference in the expression of transferrin receptor between GOLD 4 COPD lungs and non-COPD lungs (Fig. 3B).

To determine which cells in the lungs expressed transferrin and whether the expression of transferrin was associated with iron deposition, lung sections of non-COPD and GOLD 4 COPD subjects were co-stained for Perls-DAB and transferrin. Non-COPD lungs showed scant staining for transferrin, localized mainly to alveolar macrophages based on location and cell morphology (Fig. 3C). However, in GOLD 4 COPD lungs, the majority of the transferrin-positive cells were parenchymal cells, and not the iron-positive alveolar macrophages (Fig. 3D).

Together, these data suggest that iron-uptake gene expression is increased in severe COPD lungs compared to non-COPD lungs, but the iron-binding protein transferrin is not expressed by the macrophages which accumulate the iron.

### Expression of Iron Retention and Homeostasis Genes in COPD Lungs

**Figure 4:**
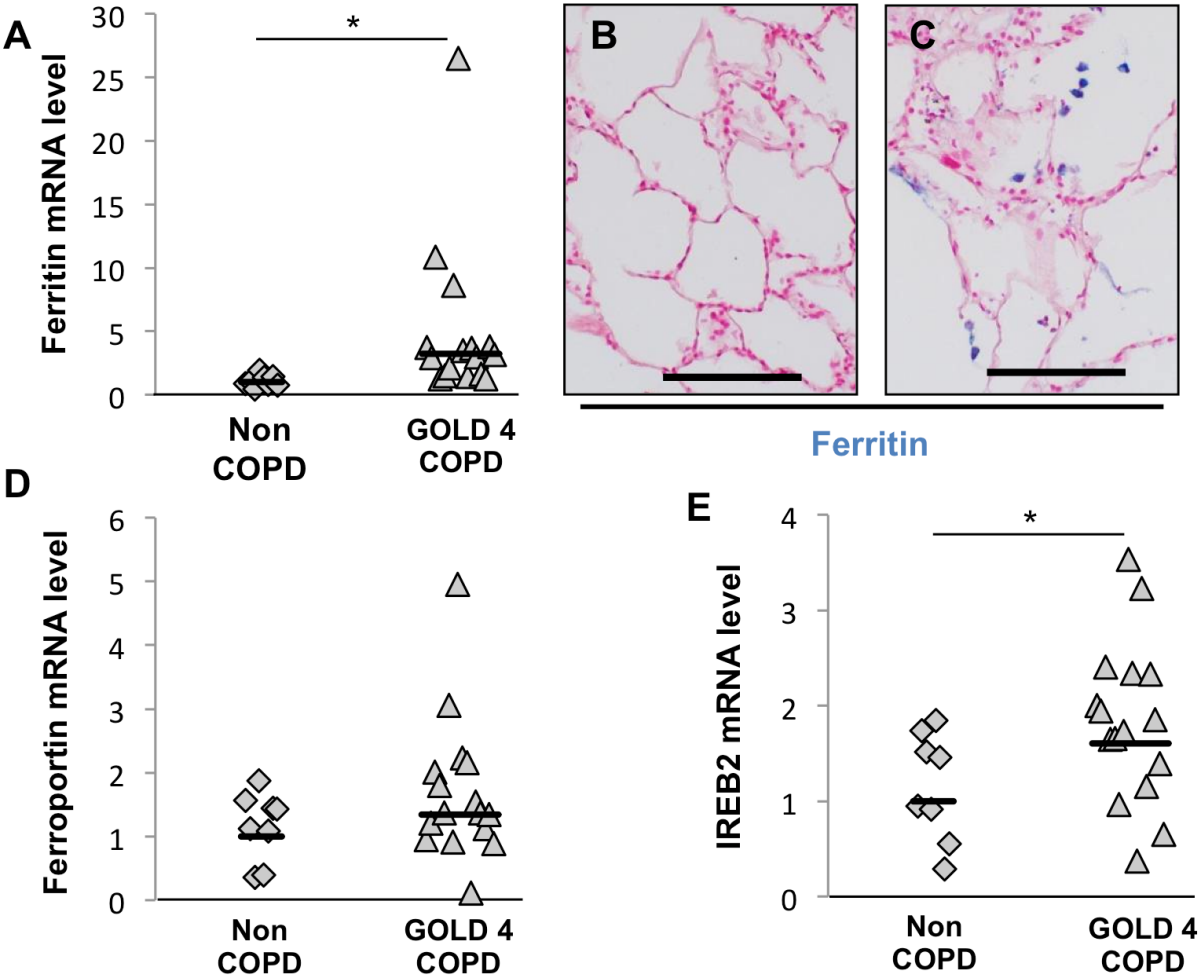
Iron storage capacities are altered in lungs of COPD subjects. mRNA expression of (A) ferritin, (D) ferroportin and (E) IREB2 was investigated in whole-lung total RNA samples obtained from 8 subjects without COPD and 16 subjects with GOLD 4 COPD.Three reference genes (GAPDH, HPRT1 and PPIA) were used for normalization. Immunohistochemistry study of ferritin, with nuclear fast red counterstaining, in lung samples obtained from (B) a subject without COPD and (C) a subject with GOLD 4 COPD. *p<0.05 (Student’s t test), Scale bars = 125 µm.

Net iron accumulation could also be caused by an increase in cellular iron retention and/or a decrease in iron export. Accordingly, we examined the expression of genes related to iron retention and export, ferritin and ferroportin, respectively, by real-time RT-PCR using RNA from 8 non-COPD lung samples and 16 GOLD 4 COPD lung samples. Ferritin mRNA expression was significantly increased in GOLD 4 COPD lungs compared to non-COPD lungs (fold increase = 3.22, p = 0.031, Fig. 4A), while the expression of ferroportin mRNA was unchanged (Fig. 4D). Consistent with the increased intracellular retention of iron in macrophages in COPD lungs, increased ferritin staining in COPD lungs (Fig. 4B) compared to non-COPD lungs (Fig. 4C) localized to alveolar macrophages. IREB2 mRNA expression was signifcantly higher in GOLD4-COPD than in non-COPD (fold increase = 1.6, p = 0.045, Fig. 4E).

We also looked for a correlation between the expression of these iron metabolism related genes and the emphysema severity. We did not find any statistically signficant correlation betwen the expression of these genes and the mean radiograph attenuation of the lung tissue (Table S2).

**Figure 5:**
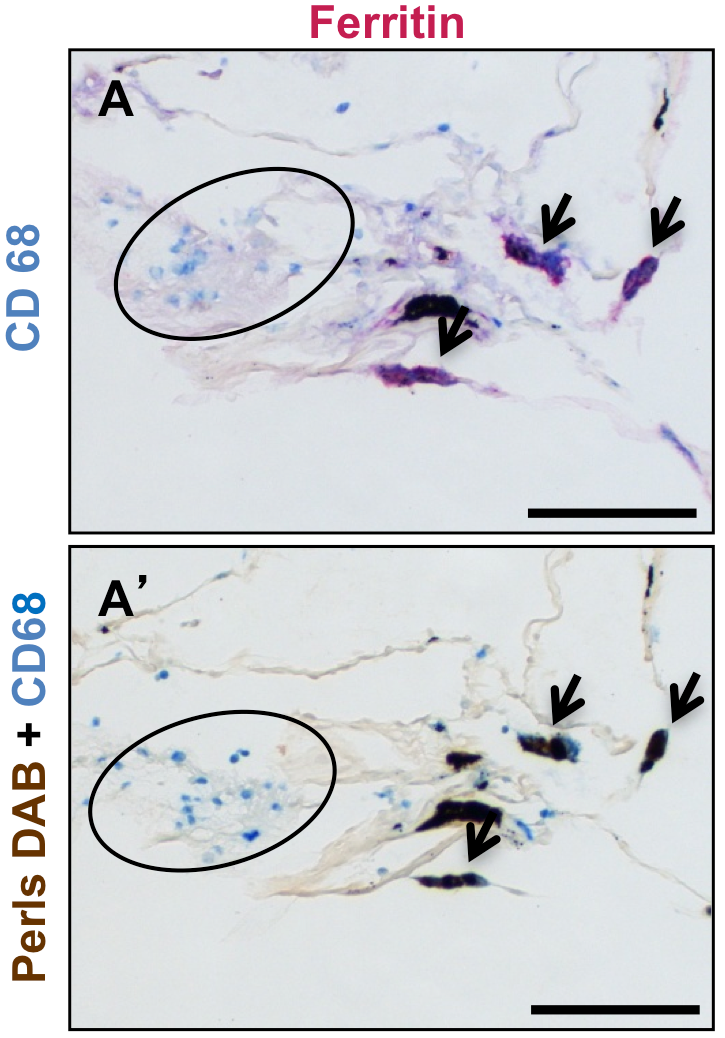
Iron deposits and ferritin staining are colocalized in the same subset of macrophages (A) Costaining for ferritin and CD68 and (A’) with Perls-DAB and CD68 in two adjacent lung sections from a GOLD 4 COPD patient. The arrows show iron and ferritin positive macrophages, inside circles are iron and ferritin negative macrophages. Scale bars = 125 µm.

To determine whether iron deposits and ferritin were present in the same macrophages in COPD lungs, we performed CD68/Perls-DAB and CD68/ferritin co-staining on consecutive GOLD 4 COPD lung sections (Fig. 5). Iron-positive macrophages exhibited strong ferritin staining (arrows). Inversely, iron-negative macrophages did not have any ferritin staining (within circle).

These data suggest that the iron accumulation in alveolar macrophages in severe COPD lungs may, at least in part, be due to an increase in cellular iron retention mechanisms.

### Expression of Iron Metabolism Genes in GOLD 1*–*3 COPD BAL Cells

**Figure 6:**
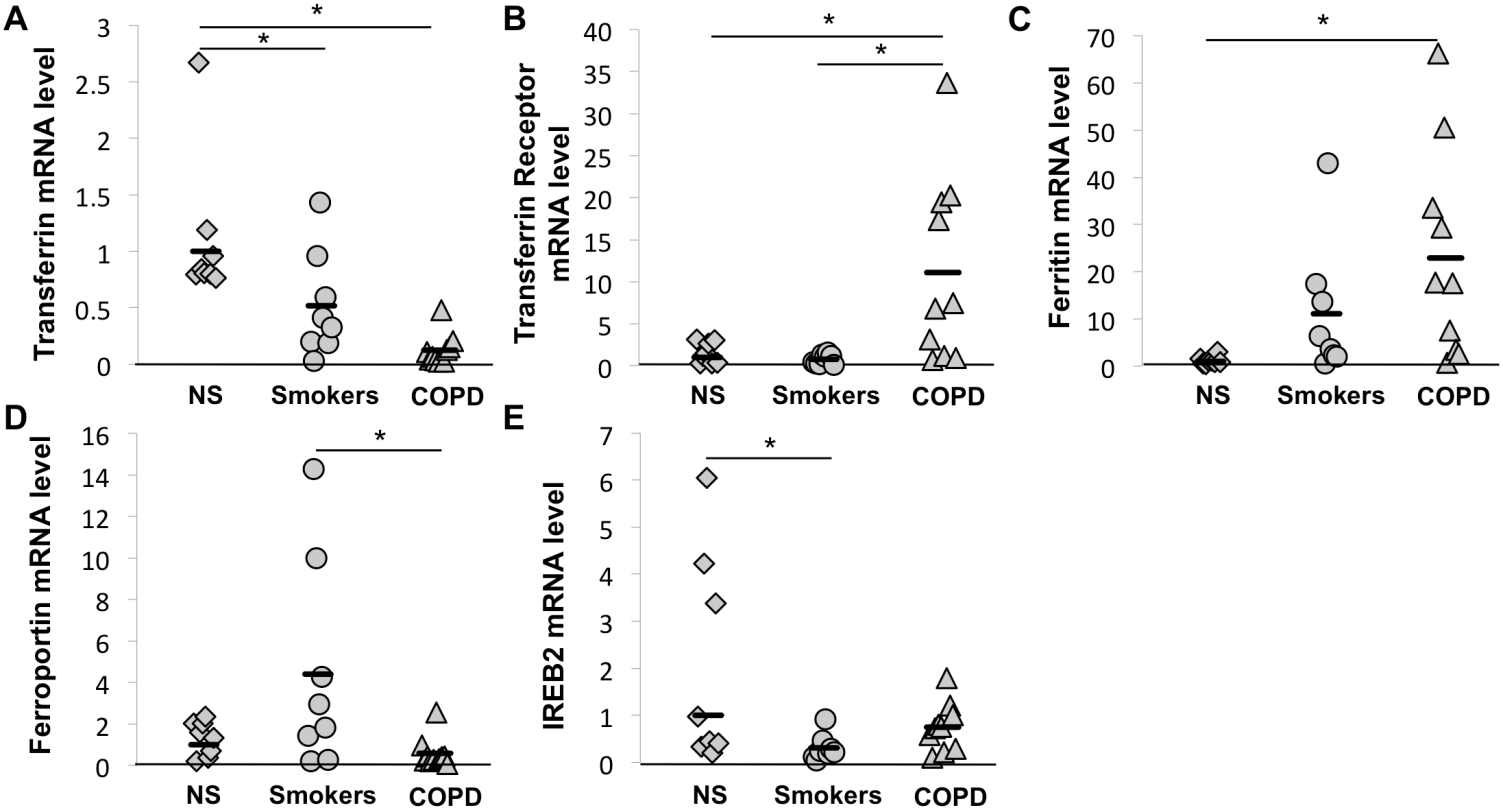
Expression of proteins involved in iron metabolism is altered in BAL cells obtained in subjects with COPD. Expression of proteins involved in iron metabolism in BAL cells collected in 8 non smokers without COPD, 8 smokers without COPD and 10 GOLD 1–3 COPD subjects was examined by RT-PCR.Three reference genes (GAPDH, HPRT1 and PPIA) were used for normalization. *p<0.05 (Anova analysis with a Tukey-Kramer post-hoc test), NS: non smokers.

The data presented in Figures 3 and 4 were obtained using mRNA from whole lung tissue samples. To better understand iron accumulationin alveolar macrophages of severe COPD lungs, we investigated the expression of iron metabolism genes in BAL cells (Fig. 6). By cytological analysis, nearly 90% of cells in the BAL fluid were macrophages (Table 2). Therefore, the data obtained from these studies may largely reflect gene expression by macrophages.

Compared to non-smokers, transferrin expression by BAL cells from non-COPD smokers and COPD subjects was significantly decreased (fold increase = 0.52 and 0.13 repectively, p = 5.7×10^−4^, Fig. 6A). In contrast, the transferrin receptor was more highly expressed in BAL cells from COPD subjects compared to non-smokers or non-COPD smokers (fold increase = 11 or 14 respectively, p = 6.7×10^−3^, Fig. 6B). When investigating mechanisms of iron retention, we found a significantly higher expression of ferritin by BAL cells from COPD subjects compared to non-smokers (fold increase = 23, p = 0.028, Fig. 6C). Interestingly, whereas ferroportin expression appeared to be similar between BAL cells from non-smokers and COPD subjects, its expression was significantly higher in BAL cells from non-COPD smokers than in the COPDsubjects (fold increase = 7.5, p = 0.028, Fig. 6D). IREB2 expression did not differ between the COPD and the other groups (Fig. 6E). These data support those presented above and show that the expression of iron metabolism genes is altered in alveolar macrophages from COPD patients compared to non-COPD patients which could result in the increased iron accumulation.

### Iron Metabolism Gene Expression in BAL Cells Correlates with Airflow Limitation

**Figure 7:**
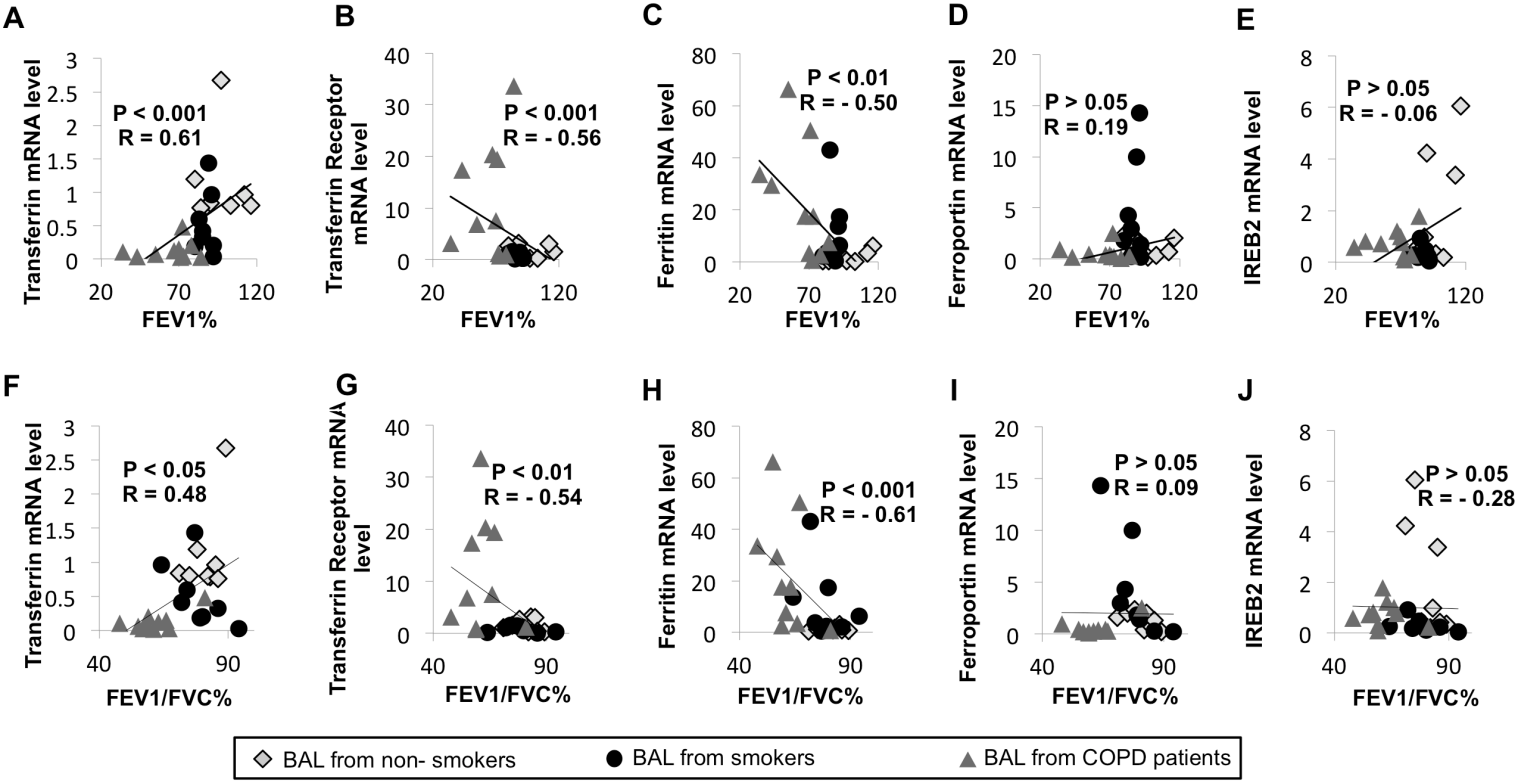
In BAL cells, expression of proteins involved in iron metabolism correlate with airflow limitation. (A–J) Correlation between the expression of genes encoding proteins involved in iron metabolism in BAL cells, as determined by RT-PCR, and airflow limitation was assessed in 8 non smokers without COPD, 8 smokers without COPD and 10 GOLD 1–3 COPD subjects.Three reference genes (GAPDH, HPRT1 and PPIA) were used for normalization. (Spearman rank correlation).

Next, we investigated correlations between expression of genes related to iron metabolism by BAL cells and airflow limitation indices (Fig. 7). Expression of transferrin positively correlated with both FEV_1_and the ratio FEV_1_/FVC (Fig. 7 A and F). Inversely, expression of the transferrin receptor and ferritin negatively correlated with airflow limitation (Fig. 7 B–G and C–H). Finally, the expression of ferroportin and IREB2 did not significantly correlate with the airflow limitation. Interestingly, expression of these iron related genes did not correlate with hemoglobin or CRP serum concentration. IREB2 expression only correlated with subject age (Table S3). Similarly, expression of studied iron related genes was not influenced by the subject sex or the presence of chronic bronchitis (Table S4). The expression of some iron metabolism related genes were associated with dyspnea severity (transferrin), exacerbation rate (transferrin and ferritin) (Table S4) and smoking history including smoking pack-year (transferrin and ferritin) (Table S3) and smoking status (transferrin, ferritin and ferroportin) (Table S5). Finally, in the COPD patients, no relation was found between the presence of an inhaled treatment and the expression of iron metabolism related genes (data not shown).

Globally, these data demonstrate that changes in macrophage expression of iron metabolism genes correlate with airflow limitation and COPD severity.

## Discussion

The main findings of this study are that: 1) iron deposits are localized in macrophages in COPD lungs; 2) the quantity of lung iron deposits increases with COPD and emphysema severity; 3) expression of transferrin (involved in iron uptake), and of ferritin (involved in iron storage), are increased in severe COPD lungs whereas ferroportin (involved in cellular excretion of iron) is unchanged; 4) in BAL cells from COPD subjects at GOLD stage 1–3, expression of transferrin receptor and ferritin expression are increased, and 5) indices of airflow limitation correlate with expression of transferrin, transferrin receptor and ferritin in BAL cells from healthy non smokers and smokers, and COPD subjects.

Consistent with our results, iron accumulation has been reported in the lungs of cigarette smokers [4]–[6] and in severe emphysema [7] but the mechanisms sustaining the iron accumulation in lungs from COPD patients have never been explored. Iron uptake, storage and sequestration are of interest in COPD. Indeed in other diseases associated with aging, including atherosclerosis, Parkinson’s and Alzheimer’s, iron depositions are postulated to contribute to excess oxidative stress [2], which is now recognized as important in the pathogenesis of COPD [20], [21]. Moreover, free iron acumulation in the lung may promote bacterial growth and influence COPD exacerbation. Therefore, the free iron pool has to be tightly controlled to protect the lung against the harmful properties of iron.

Iron bound by transferrin is taken up into cells by the transferrin receptor, and is stored in cells bound to ferritin. Compared to control lungs, higher transferrin and ferritin expression was found in COPD lungs, and transferrin receptor was higher in BAL from COPD subjects than in healthy subjects. Further, the expression of ferroportin, the only known iron exporter, was unchanged with COPD. These findings support active iron sequestration by alveolar macrophages in COPD lungs, and may represent a protective maneuver intended to control free iron, and therefore, perverse effects of iron. In fact, O. Olakanmi et al have reported that iron sequestration by alveolar macrophages decresease the formation of the highly toxic hydroxyl radical [22] and more recently, it has been shown that iron sequestration by macrophages protects A549 cells against iron toxicity [23]. Consistent with these resluts, we did not find a spatial relationship between the accumulation of 8-hydroxyguanosine, a marker of nucleic acids oxidation, and iron staining in COPD lung specimens in this study (data not shown). While not conclusive, this suggests at least that iron accumulation process in alveolar macrophages does not contribute locally to increased oxidative stress and may even decrease iron induced oxidative stress. Interestingly, current cigarette smoke exposure was not found necessary to alter iron metabolism. Indeed, GOLD 4 COPD subjects in this study had ceased cigarette smoking for at least 6 months prior to transplant and processing of their lung tissue.

The clinical relevance of these findings is supported by the correlations between expression levels of several iron pathway mRNAs in BAL cells and indices of airflow limitation in this study. Ferritin and transferrin receptor expression were increased in COPD subjects and correlated with a decrease in FEV_1_ or FEV_1_/FVC ratio. IREB2 expression tended to be higher in BAL of COPD subjects and correlated negatively with FEV_1_/FVC ratio, supporting a shift in iron metabolism in macrophages in COPD. These studies were limited to smokers, former smokers, and GOLD 2 and -3 COPD subjects, as there is high risk in obtaining BAL from GOLD 4 COPD subjects. Another aspect of the study which may be a limitation is that distinct study sets were employed for the tissue-based and BAL-based experiments. Alternatively, that iron metabolism was altered in separate cohorts of subjects may lend greater weight to the findings. Altering lung iron uptake in macrophages during cigarette smoke exposure in animal model could be employed to test whether this impacts overall lung oxidative stress and the progression of alveolar enlargement. In vitro studies in macrophages could further test the relationships between cigarette smoke exposure, free iron, iron sequestration, ROS generation and oxidative stress.

Overall this study demonstrates that macrophages in the lungs of COPD subjects have increased iron uptake and storage, likely through increased expression of transferrin, transferrin receptor and ferritin, while ferroportin expression is unchanged. This should result in a net gain in iron sequestration in COPD lung macrophages, which we postulate is a protective mechanism intended to reduce free iron and its harmful effects. Similar to the robust anti-oxiodant response reported in COPD lungs [21], it seems likely that iron sequestration may be a mechanism that is intended to limit, but fails to eliminate, progression of COPD. Further investigations are needed to elucidate the contribution of iron sequestration in alveolar macrophages in the complex pathophysiology of COPD.

## Supporting Information

Table S1 Sequence of primer pairs used in our RT-PCR reactions.
(DOCX)

Table S2 Lack of relationship between subject lung density in CT Hounsfield units and iron metabolism gene expression.
(DOCX)

Table S3 Relationships between expression of iron metabolism-related mRNAs age, smoking pack years, and KCO, but not serum variables.
(DOCX)

Table S4 Lack of relationship between expression of iron metabolism-related mRNAs and subject gender, dyspnea. Transferrin correlate with the presence of chronic bronchitis. Transferrin and ferritin are correlated with the presence of exacerbations.
(DOCX)

Table S5 Expression of transferrin, ferritin and IREB2 are impacted by smoking status.
(DOCX)
