# Supplementary material for: Increased Iron Sequestration in Alveolar Macrophages in Chronic Obtructive Pulmonary Disease
Source: PLoS One. 2014 May 1;9(5):e96285. doi: 10.1371/journal.pone.0096285 (PMC4006868; doi:10.1371/journal.pone.0096285)
Supplement: Table S1 — Sequence of primer pairs used in our RT-PCR reactions. (DOCX) [file pone.0096285.s001.docx]

| **Gene** | **Sequences 5'-> 3'** | |
| --- | --- | --- |
| **Ferritin** | *Forward* | CCGCAGGGCCAGACGTTCTT |
|  | *Reverse* | CCGGGTTCCGTCCAAGCACTG |
| **Transferrin** | *Forward* | TGCCAGAGTTTCCGCGACCA |
|  | *Reverse* | TTCGTTTGCCGCAATGGCCC |
| **IREB2** | *Forward* | GCGATGGACGCCCCAAAAGC |
|  | *Reverse* | AGGCAGAACATCATACTTGGTGCC |
| **Ferroportin** | *Forward* | GAGCAGCAGCAGCGATAG |
|  | *Reverse* | AGAATGACCAAGGTAGAGAAGG |
| **Transferrin receptor** | *Forward* | AAAATCCGGTGTAGGCA |
|  | *Reverse* | TTAAATGCAGGGACGAAAGG |
| **GAPDH** | *Forward* | TGCACCACCAACTGCTTAGC |
|  | *Reverse* | GGCATGGACTGTGGTCATGAG |
| **HPRT** | *Forward* | GACCAGTCAACAGGGGACAT |
|  | *Reverse* | AACACTTCGTGGGGTCCTTTTC |
| **PPIA** | *Forward* | GGCAAATGCTGGACCCAACACA |
|  | *Reverse* | TGCTGGTCTTGCCATTCCTGGA |

**Table S1:** **Sequence of primer pairs used in our RT-PCR reactions.**
