# Supplementary material for: Increased Iron Sequestration in Alveolar Macrophages in Chronic Obtructive Pulmonary Disease
Source: PLoS One. 2014 May 1;9(5):e96285. doi: 10.1371/journal.pone.0096285 (PMC4006868; doi:10.1371/journal.pone.0096285)
Supplement: Table S2 — Lack of relationship between subject lung density in CT Hounsfield units and iron metabolism gene expression. (DOCX) [file pone.0096285.s002.docx]

|  | **Correlation between HU and expression of:** | | | | |
| --- | --- | --- | --- | --- | --- |
|  | Transferrin | Transferrin Receptor | Ferritin | Ferroportin | IREB 2 |
| R^2^ | 0.27 | 0.24 | 0.20 | 0.47 | 0.33 |
| **P-value1** | **0.28** | **0.33** | **0.44** | **0.06** | **0.19** |

*^1^*Spearman Rho; *^2^* P-value of Spearman rank correlation; HU: Hunsfield Unit (CT scan)

**Table S2: Lack of relationship between subject lung density in CT Hounsfield units and iron metabolism gene expression.**
