# Supplementary material for: Increased Iron Sequestration in Alveolar Macrophages in Chronic Obtructive Pulmonary Disease
Source: PLoS One. 2014 May 1;9(5):e96285. doi: 10.1371/journal.pone.0096285 (PMC4006868; doi:10.1371/journal.pone.0096285)
Supplement: Table S3 — Relationships between expression of iron metabolism-related mRNAs age, smoking pack years, and KCO, but not serum variables. (DOCX) [file pone.0096285.s003.docx]

|  | **Variable** | | | | | | | | | |  |
| --- | --- | --- | --- | --- | --- | --- | --- | --- | --- | --- | --- |
|  | **Serum Hemoglobin** | | **Serum CRP** | | **Subject Age** | | **Smoking Pack-Year** | | **KCO** | |  |
|  | *R^1^* | *P value^2^* | *R^1^* | *P value^2^* | *R^1^* | *P value^2^* | *R^1^* | *P value^2^* | *R^1^* | *P value^2^* |  |
| **Transferrin** | | - 0.011 | 0.96 | -0.14 | 0.52 | - 0.13 | 0.53 | - 0.43 | ***0.030*** | O.43 | ***0.046*** |
| **Transferrin Receptor** | | 0.11 | 0.61 | -0.067 | 0.75 | 0.27 | 0.19 | 0.059 | 0.77 | 0.015 | 0.95 |
| **Ferritin** | | - 0.13 | 0.55 | 0.007 | 0.97 | 0.18 | 0.39 | 0.535 | ***0.0049*** | -0.11 | 0.63 |
| **Ferroportin** | | - 0.13 | 0.54 | -0.086 | 0.68 | - 0.098 | 0.63 | - 0.004 | 0.99 | 0.32 | 0.15 |
| **IREB2** | | - 0.23 | 0.26 | -0.16 | 0.45 | 0.55 | ***0.004*** | - 0.21 | 0.29 | 0.29 | 0.19 |

*^1^*Spearman Rho; *^2^* P-value of Spearman rank correlation.

**Table S3: Relationships between expression of iron metabolism-related mRNAs age, smoking pack years, and KCO, but not serum variables.**
