# Supplementary material for: Increased Iron Sequestration in Alveolar Macrophages in Chronic Obtructive Pulmonary Disease
Source: PLoS One. 2014 May 1;9(5):e96285. doi: 10.1371/journal.pone.0096285 (PMC4006868; doi:10.1371/journal.pone.0096285)
Supplement: Table S4 — Lack of relationship between expression of iron metabolism-related mRNAs and subject gender, dyspnea. Transferrin correlate with the presence of chronic bronchitis. Transferrin and ferritin are correlated with the presence of exacerbations. (DOCX) [file pone.0096285.s004.docx]

|  |  | **Relative expression of:** | | | | |
| --- | --- | --- | --- | --- | --- | --- |
|  | *Number* | Transferrin | Transferrin Receptor | Ferritin | Ferroportin | IREB2 |
| F.I. Female vs. Male | 9 / 17 | 1.1 | 1.0 | 2.5 | 1.1 | 0.68 |
|  | **P value^1^** | **0.85** | **0.98** | **0.12** | **0.92** | **0.51** |
| F.I Dyspnea mMRC 0-1 vs. 2-3 | 16 / 10 | 0.37 | 1.45 | 2.40 | 0.44 | 0.65 |
|  | **P value^1^** | **0.04*** | **0.57** | **0.18** | **0.14** | **0.54** |
| F.I. No chronic bronchitis vs. chronic bronchitis | 17 / 9 | 0.43 | 1.51 | 3.78 | 1.75 | 0.41 |
|  | **P value^1^** | **0.06** | **0.51** | **0.05** | **0.48** | **0.10** |
| F.I. No exacerbation vs. ≥1 exacerbation per year | 15/9 | 0.3 | 4.1 | 5.7 | 1.1 | 0.8 |
|  | **P value^1^** | **0.024*** | **0.092** | **0.017*** | **0.915** | **0.628** |

^1^Student's t test, F.I.: Fold Increased

**Table S4: Lack of relationship between expression of iron metabolism-related mRNAs and subject gender, dyspnea. Transferrin correlate with the presence of chronic bronchitis. Transferrin and ferritin are correlated with the presence of exacerbations.**
