# Supplementary material for: Increased Iron Sequestration in Alveolar Macrophages in Chronic Obtructive Pulmonary Disease
Source: PLoS One. 2014 May 1;9(5):e96285. doi: 10.1371/journal.pone.0096285 (PMC4006868; doi:10.1371/journal.pone.0096285)
Supplement: Table S5 — Expression of transferrin, ferritin and IREB2 are impacted by smoking status. (DOCX) [file pone.0096285.s005.docx]

|  |  | **Relative expression of:** | | | | |
| --- | --- | --- | --- | --- | --- | --- |
| *Smoking Status* | *Number* | Transferrin | Transferrin Receptor | Ferritin | Ferroportin | IREB2 |
| Never Smoke | 8 | 1.0 | 1.0 | 1.0 | 1.0 | 1.0 |
| Former Smoker | 4 | 3.3 | 0.27 | 26 | 0.66 | 0.35 |
| Current Smoker | 14 | 0.5 | 1.7 | 12 | 2.0 | 0.26 |
|  | **P value^1^** | **0.0023*** | **0.26** | **0.014*** | **0.499** | **0.048*** |

^1^Anova analysis

**Table S5: Expression of transferrin, ferritin and IREB2 are impacted by smoking status.**
